# Progressive inflammation reduces high-frequency EEG activity and cortical dendritic arborisation in late gestation fetal sheep

**DOI:** 10.1186/s12974-023-02805-x

**Published:** 2023-05-24

**Authors:** Sharmony B. Kelly, Justin M. Dean, Valerie A. Zahra, Ingrid Dudink, Alison Thiel, Graeme R. Polglase, Suzanne L. Miller, Stuart B. Hooper, Laura Bennet, Alistair J. Gunn, Robert Galinsky

**Affiliations:** 1 The Ritchie Centre, Hudson Institute of Medical Research, 27-31 Wright Street, Melbourne, VIC 3168 Australia; 2 Department of Obstetrics and Gynaecology, Monash University, Melbourne, VIC Australia; 3 Department of Physiology, The University of Auckland, Auckland, New Zealand

**Keywords:** Neuroinflammation, Neurodevelopment, Fetal infection/inflammation, Neurons

## Abstract

**Background:**

Antenatal infection/inflammation is associated with disturbances in neuronal connectivity, impaired cortical growth and poor neurodevelopmental outcomes. The pathophysiological substrate that underpins these changes is poorly understood. We tested the hypothesis that progressive inflammation in late gestation fetal sheep would alter cortical neuronal microstructure and neural function assessed using electroencephalogram band power analysis.

**Methods:**

Fetal sheep (0.85 of gestation) were surgically instrumented for continuous electroencephalogram (EEG) recording and randomly assigned to repeated saline (control; *n* = 9) or LPS (0 h = 300 ng, 24 h = 600 ng, 48 h = 1200 ng; *n* = 8) infusions to induce inflammation. Sheep were euthanised 4 days after the first LPS infusion for assessment of inflammatory gene expression, histopathology and neuronal dendritic morphology in the somatosensory cortex.

**Results:**

LPS infusions increased delta power between 8 and 50 h, with reduced beta power from 18 to 96 h (*P* < 0.05 vs. control). Basal dendritic length, numbers of dendritic terminals, dendritic arborisation and numbers of dendritic spines were reduced in LPS-exposed fetuses (*P* < 0.05 vs. control) within the somatosensory cortex. Numbers of microglia and interleukin (IL)-1β immunoreactivity were increased in LPS-exposed fetuses compared with controls (*P* < 0.05). There were no differences in total numbers of cortical NeuN + neurons or cortical area between the groups.

**Conclusions:**

Exposure to antenatal infection/inflammation was associated with impaired dendritic arborisation, spine number and loss of high-frequency EEG activity, despite normal numbers of neurons, that may contribute to disturbed cortical development and connectivity.

## Introduction

Exposure to antenatal infection/inflammation is a leading cause of brain injury and neurodevelopmental impairment [1]. For example, exposure to infection/inflammation increases the risk of cerebral palsy by 2- to 12-fold in near-term/term infants [2, 3]. Improving our understanding of the pathogenesis of brain injury is essential for better injury detection and development of interventions to improve outcomes, and reduce the socioeconomic burdens on the affected individuals, their families and society [4].

Perinatal infection/inflammation is common in low, middle and high-income countries [5], and is associated with grey matter abnormalities. For example, neuroimaging studies have shown that chorioamnionitis is independently associated with reduced sulcal depth and cortical volume in the temporal lobe, without overt grey matter injury [6, 7].

In rodents, chorioamnionitis induced with a single intrauterine injection of lipopolysaccharide (LPS) was associated with reduced dendritic arborisation in cortical neuronal cultures [8]. In pregnant rat dams, twelve hourly intraperitoneal (i.p.) injections of inactivated group B *Streptococcus* (10^9^ CFU) between G19 and G22 (term) led to cortical thinning and neuromotor impairments in the offspring at postnatal day 40 [9]. Similarly, mild-to-moderate inflammation in neonatal rats using daily i.p. LPS (0.3 mg/kg) injections from P1–3 reduced dendritic arborisation and spine formation in cortical pyramidal neurons without overt neuronal loss [10].

While these preclinical data provide a compelling link between acute inflammation and impaired cortical development, the most common clinical fetal and neonatal scenario involves a progressive inflammatory response without severe fetal compromise [11]. Consistent with this, in fetal sheep live bacterial inoculation is associated with progressive systemic inflammation [12]. Similarly, recent clinical evidence strongly suggests that antenatal inflammation is a progressive process that persists into the newborn period [13] and supports the concept that sustained inflammation is associated with long-term impairments in brain development [14–17].

It is important to find ways to rapidly and non-invasively identify such progressive inflammation-induced injury at the bedside. Electroencephalography (EEG) is widely used to identify functional impairments in cases of preterm and term encephalopathy [18, 19]. However, the relationship between EEG and abnormal development of the neuronal microstructure is poorly understood. Thus, in the present study we tested the hypothesis that progressive antenatal inflammation induced by repeated, increasing-dose infusions of Gramnegative LPS would be associated with cortical inflammation and altered neuronal microstructural development. Furthermore, we hypothesised that pathological changes associated with altered cortical neuronal microstructural development could be detected using EEG assessment of neural function.

## Materials and methods

All procedures were approved by the Hudson Institute of Medical Research Animal Ethics committee and were conducted in accordance with the National Health and Medical Research Council Code of Practice for the Care and Use of Animals for Scientific Purposes (Eighth Edition). The experiments are reported in accordance with the ARRIVE guidelines for reporting animal research [20]. In this study, we compared two groups of interest: (i) vehicle controls and (ii) antenatal inflammation. The key outcome measures were: (i) cortical inflammation, (ii) neuronal microstructural development, assessed using dendritic length, numbers of dendritic terminals, neuronal arborisation and numbers of dendritic spines and (iii) EEG measures of neuronal function. Seventeen pregnant Border-Leicester ewes bearing singleton or twin fetuses underwent aseptic surgery at either 124 or 125 days of gestation. Food but not water was withdrawn approximately 18 h before surgery. Anaesthesia was induced by i.v injection of sodium thiopentone (20 mL) and maintained using 2–3% isoflurane in oxygen (Bomac Animal Health, New South Wales, Australia). Ewes received prophylactic antibiotics (ampicillin: 1 g i.v; Austrapen, Lennon Healthcare, St. Leonards, NSW, Australia, and engemycin: 500 mg i.v; Schering-Plough, Upper Hutt, New Zealand) immediately before surgery. Isoflurane levels, heart rate, oxygen saturation, and respiratory rate were continuously monitored throughout surgery by trained anaesthetic staff.

### Fetal instrumentation

A midline maternal laparotomy was performed, the fetus was exposed and partially removed from the uterus for implantation of polyvinyl catheters into the right brachiocephalic artery and amniotic cavity. In twin pregnancies, only one twin was instrumented. Two pairs of electroencephalograph (EEG) electrodes (AS633-7SSF; Cooner Wire, Chatsworth, CA, USA) were placed through burr holes onto the dura over the parasagittal parietal (somatosensory) cortex (10 and 20 mm anterior to bregma, and 10 mm lateral) and secured using surgical bone wax and cyanoacrylate glue. A catheter was inserted into the left fetal axillary vein for administration of post-operative antibiotics and lipopolysaccharide (LPS) or vehicle (saline). The fetus was returned to the uterus in its original orientation and all fetal leads were exteriorised through the maternal flank. A catheter was inserted into the maternal jugular vein for administration of post-operative antibiotics and euthanasia at the end of the experimental period. At the completion of surgery, ewes received fentanyl for 3 days via a transdermal patch placed on the left hind leg (75 μg/h; Janssen Cilag, North Ryde, NSW, USA).

Ewes were housed together in separate pens in a temperature controlled (20 ± 2 °C and relative humidity of 50 ± 10%) room with a 12-h light–dark cycle with access to food and water ad libitum. Four to five days of post-operative recovery were allowed before experiments commenced. Ewes and fetuses received daily i.v. infusions of ampicillin (800 mg, maternal i.v. and 200 mg, fetal i.v.) and engemycin (500 mg, maternal i.v.) for three consecutive days after surgery. Catheters were maintained patent with a continuous infusion of heparinised saline (25 IU/mL) at a rate of (0.2 mL/h).

### Experimental recordings

Fetal EEG was continuously recorded from 24 h prior to the first saline or LPS infusion (129 days of gestation) until the end of the experiment (134 days of gestation). The analogue fetal EEG signal was bandpass-filtered with a cut-off frequency set at 1 and 22 Hz and digitised at a sampling frequency of 400 Hz. EEG power was derived from the analogue signal, whilst spectral edge was calculated as the frequency below which 90% of the intensity was present. Relative (%) spectral power in the Δ (0–3.9 Hz), *θ* (4–7.9 Hz), *ɑ* (8–12.9 Hz), and *β* (13–22 Hz) frequency bands were quantified. This involved calculating power spectra, by fast Fourier transform, of the EEG on sequential epochs using a 10-s Hanning window to minimise spectral leakage, as previously described [21, 22].

### Experimental protocol

Experiments started at 129 days of gestation (term is ~ 147 days). This study examined 0.85 gestation fetal sheep, at an age when brain development is broadly equivalent to that of a near term/term human infant [23].

Fetuses were randomly allocated to two groups: vehicle (saline, *n* = 9 [6 males, 3 females]) or LPS (*Escherichia coli*, O55:B5, MilliporeSigma, MO, USA; *n* = 8 [6 males, 2 females]). Fetuses received 300 ng, 600 ng, and 1200 ng infusions of LPS diluted in 2 mL of saline i.v. (infusion rate: 1 mL/min) at 0, 24 and 48 h, respectively, along with a 3 mL vehicle (saline) infusion at a rate of 0.75 mL/h starting 1 h after the saline infusion, as previously described [24]. Control and LPS groups received the 3 mL vehicle infusion. Inflammation was confirmed based on increased plasma cytokine concentrations relative to baseline after LPS infusion. Serial cytokine measurements have been previously reported in Kelly et al*.* [24]. This model is relevant to the fetal inflammatory response syndrome caused by chorioamnionitis and reproduces the acute inflammatory exacerbations associated with adverse neurodevelopment [25, 27, 27]. Controls received an equivalent volume of saline at the same infusion rate. Randomisation was stratified by cohort to control for the time of year and twin pregnancy. Fetal preductal arterial blood samples were collected every morning (0900 h) starting from 30 min before the start of the experiment until the day of post-mortem for pH, blood gases, and glucose and lactate concentrations (ABL 90 Flex Plus analyser; Radiometer, Brønshøj, Denmark). Four days after the start of infusions, sheep were euthanised by intravenous injection of pentobarbitone sodium (100 mg/kg, Lethabarb, Virbac, NSW, Australia).

### Brain collection and processing

At post-mortem the right hemisphere was immersion-fixed with 10% phosphate-buffered formalin for 3 days before processing and embedding using a standard paraffin tissue preparation. Using a brain mould, the right hemisphere was cut with a blocking blade into 5-mm-thick coronal blocks. Blocks from the forebrain, approximately 23 mm anterior to stereotaxic zero, with a clearly visible lateral gyrus containing the somatosensory cortex were sectioned into 8-μm-thick coronal sections using a microtome (Leica Microsystems, Victoria, Australia).

Region matched tissue sections from the left hemisphere were rinsed in distilled water and immersion-fixed using a commercially available FD Rapid Golgi Stain Kit (FD Neurotechnologies Inc., MD, USA). Tissue containing a clearly visible lateral gyrus containing the somatosensory cortex was frozen and sectioned with a Leica VT1200S vibratome at 100 μm. The sections were mounted onto coverslips, processed for Golgi visualisation, dehydrated in a graded series of alcohol solutions and cover slipped.

### Gene expression analysis

Remaining grey matter tissue from the lateral gyrus, adjacent to the section collected for Golgi staining, was dissected, snap-frozen in liquid nitrogen and stored at − 80 °C for mRNA analysis of inflammatory genes. The tissue was homogenised and total mRNA was isolated using an RNeasy Midi Kit (QIAGEN, Venlo, Netherlands) and reverse transcribed into single stranded cDNA (SuperScript III First-Strand Synthesis System, Invitrogen, MA, USA). Relative mRNA expression levels of interleukin (IL)1A, IL1B, and IL6 were measured by qRT-PCR using an Applied Biosystems Quantstudio 6 Real-Time PCR system. Relative mRNA levels of the genes of interest were normalised to the 18S RNA for each sample by subtracting the cycle threshold (Ct) value for 18S from the Ct value for the gene of interest (ΔCt). mRNA levels of genes of interest were normalised using the formula 2 − ΔCt and the results expressed as a fold change from control. A threshold value (Ct) for each sample was measured in triplicate and a control sample containing no cDNA template was included in each run. Details of the primers used are presented in Table 1.

**Table 1:** Primer sequences for qPCR

Gene	Species	Accession number	Primer sequence	Amplicon length, nt
*18S*	Rat	NR_046237.1	5ʹ-GTAACCCGTTGAACCCCATT-3ʹ 3ʹ-CCATCCAATCGGTAGTAGCG-5ʹ	151
*IL1A*	Sheep	NM_001009808.1	5ʹ-GTCCATACATGACGGCTGCTA-3ʹ 3ʹ-GGTGTCTCAGGCATCTCCTTAT-5ʹ	184
*IL1B*	Sheep	NM_001009465.2	5ʹ-CGATGAGCTTCTGTGTGATG-3ʹ 3ʹ-CTGTGAGAGGAGGTGGAGAG-5ʹ	121
*IL6*	Sheep	NM_001009392	5ʹ-CGCAAAGGTTATCATCATCC-3ʹ 3ʹ CCCAGGAACTACCACAATCA-5ʹ	108

### Immunohistochemistry

Slides were baked at 60 °C for 1 h then dewaxed in xylene, rehydrated in increasing concentrations of ethanol and washed in 0.1 mol/L phosphate buffered saline (PBS). Antigen retrieval was performed in citrate buffer (pH 6) using a microwave for 15 min. Endogenous peroxide quenching was performed by incubating slides in 0.1% H_2_O_2_ in methanol. Non-specific antigen blocking was performed using 3% normal goat serum. Sections were labelled with 1:250 rabbit anti-IL-1β (cat#: NB600-633, Novus, CO, USA), 1:200 rabbit anti-ionised calcium binding adaptor molecule 1 (Iba-1, cat#: ab153696, Abcam, Cambridge, UK) 1:200 rabbit anti-glial fibrillary acidic protein (GFAP; cat#: ab68428, Abcam) 1:350 rabbit anti-neuronal nuclei (NeuN, cat#: ab177487, Abcam), and 1:800 rabbit anti-Caspase3 (cat#: AF835, R&D Systems, MS, USA), overnight at 4 °C. Sections were incubated in biotin conjugated IgG goat anti-rabbit (1:200; Dako, Victoria, Australia) for 3 h at room temperature before being incubated in avidin–biotin complex (MilliporeSigma) for 45 min at room temperature. Sections were reacted with 3,3’-diaminobenzidine tetrahydrochloride (MilliporeSigma). The reaction was stopped in PBS and slides were then dehydrated in xylene followed by increasing concentrations of ethanol, mounted in dibutyl phthalate polystyrene xylene and cover slipped.

ApopTag was used to detect single and double-stranded breaks in DNA associated with apoptosis [28]. Staining was carried out according to manufacturer’s instructions (MilliporeSigma, s7100, ApopTag Peroxidase in Situ Apoptosis Detection Kit). In brief, tissue was dewaxed in xylene, rehydrated in increasing concentrations of ethanol, and washed in PBS. The tissue was then pre-treated with proteinase K for 15 min, washed in PBS, and background peroxidase activity quenched in 3.0% hydrogen peroxide for 5 min. The equilibration buffer was added for 10 s, before the TdT enzyme was added and left for 1 h at 37 °C. The reaction was stopped in stop buffer for 10 min, then washed before adding the anti-digoxigenin conjugate for 30 min at room temperature. Finally, peroxidase substrate was added for 6 min before the tissue was counterstained in 50% haematoxylin (cat#MH-1NPR, Trajan Scientific, VIC, Australia), dehydrated in xylene followed by increasing concentrations of ethanol, mounted in dibutyl phthalate polystyrene xylene and cover slipped.

### Immunohistochemistry analysis

**Fig. 1:**
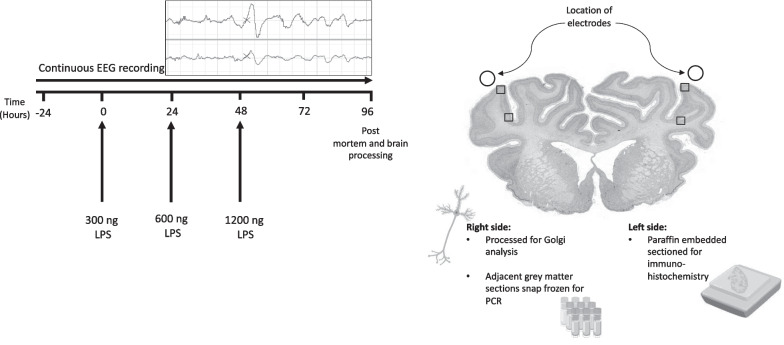
Schematic outlining the study design. The study consisted of two groups: control (vehicle, *n* = *9*) and LPS (*n* = *8*). The solid lines show the timing of the lipopolysaccharide (LPS)/vehicle infusions which were given over 2 min at increasing doses (300 ng, 600 ng, and 1200 ng). Controls received an equivalent volume of vehicle (saline) during the infusion period. Continuous electroencephalogram (EEG) recordings were performed throughout the experiential period. At 96 h, brains were collected for Golgi staining to examine neuronal arborisation and numbers of dendritic spines, immunohistochemistry to assess neuroinflammation, neuronal numbers and cortical area and mRNA assessment of proinflammatory proteins. Boxes indicate regions of interest used for immunohistochemical and Golgi analysis. Image source:[86]

Cortical areas were quantified using QuPath imaging software (version 0.2.3) [29]. Microglia (Iba-1 + cells), neuronal nuclei (NeuN + cells), and caspase 3 + cells were visualised using light microscopy (Olympus, Tokyo, Japan) at 40 × magnification using CellSens imaging software (version 2.3; Olympus). Caspase 3 + cells displaying both immunostaining and apoptotic bodies were counted. NeuN + cells were counted only if they were morphologically normal, while cells displaying condensed or fragmented nuclei were not counted [30]. IL-1β-stained sections were scored at 20 × magnification using an immunoreactivity scoring system adapted from Girard et al. [31]. Scoring was based on the intensity of staining whereby 1 = light, 2 = moderate, 3 = moderate to intense and 4 = intense, as previously reported [32]. The area fraction of GFAP (astrocyte) staining was quantified in ImageJ software (v2.0, LOCI, University of Wisconsin) using a standard intensity threshold. ApopTag + cells were quantified using QuPath imaging software. Total numbers of positive cells were counted within the lateral gyrus from the parietal lobe between cortical layers 3 and 5. For all other immunohistochemical analyses, positive cells or immunoreactivity were quantified for each field of view (1 field from the base of the gyrus and 1 field from the top of the gyrus; Fig. 1) from two sections per subject using ImageJ. For each field of view, average scores from two slides from the right hemisphere were calculated. The size of the field of view was the same for all assessments (20 x magnification = 0.489 mm^2^ and 40 x magnification = 0.130 mm^2^). All imaging and cell counts were performed by an assessor who was blinded to the treatment group by independent coding of slides and data files.

### Golgi analysis

Coded region-matched Golgi-stained tissue sections (10 serial sections per subject) were used to assess basal dendrites from pyramidal neurons in layers 3 and 5 of the lateral cortex as described previously [33, 34]. The Golgi staining tended to produce less complete filling of the apical dendrites relative to the basal dendrites. Thus, apical dendrites were not analysed. Basal dendrites were visualised using an Olympus BX61 stereology microscope (Olympus) equipped with an Olympus DP73 colour camera (× 0.5 lens) at 60 × magnification and CellSens imaging software (version 2.3; Olympus). From each subject, basal dendrites from a total of 20 pyramidal neurons selected from 10 serial sections of the lateral gyrus met the inclusion criteria for imaging. A total of 10 neurons were selected from the base of the gyrus and 10 were selected from the top of the gyrus (Fig. 1). We found that 20 was the maximum number of neurons per subject that could be selected based on the pre-defined selection criteria. Neurons were selected based on morphological criteria [34]: triangular shaped soma and apical dendrites perpendicular to the pial surface, complete Golgi impregnation of the cell that permitted visualisation of the entire dendritic arbour and spines, neuronal soma and processes not obscured by other neurons, glia or blood vessels, and neurons exhibiting a complete basilar dendritic tree without truncated or cut processes. Pyramidal neuronal subtypes were not distinguished. A preliminary analysis of *n* = 4 subjects per group showed no significant differences between neurons selected from the base of the gyrus and neurons selected from the top of the gyrus for dendritic length (control: gyrus base = 2874 ± 495, gyrus top = 3261 ± 517; LPS: gyrus base = 1663 ± 137, gyrus top = 1945 ± 173) and numbers of terminals (control: gyrus base = 42 ± 2, gyrus top = 48 ± 3; LPS: gyrus base = 30 ± 3, gyrus top = 32 ± 2). Nevertheless, equal numbers of neurons from the gyrus base (*n* = 10) and top (*n* = 10) were analysed from each subject to ensure our data were not confounded by potential regional differences in neuronal development. Images were cropped and separated into individual channels using ImageJ. The images were then imported into Imaris (version 9.2.1, Bitplane, Oxford Instruments Company, Abington, UK). Measures of dendritic complexity including summated dendritic length, numbers of dendritic terminals and Sholl analysis (numbers of dendrite intersections per Sholl ring) were assessed using the Imaris filament tracer tool. Sholl intersections were analysed using 5 μm interval concentric rings centred on the soma. Numbers of dendritic spines were quantified using the Imaris filament tracer and dendritic spine classification (filopodia, long thin, stubby, mushroom) was performed using the MATLAB spine classification extension (MATLAB, R2019a, Mathworks Inc., CA, USA).

### Data analysis and statistics

Offline analysis of physiological data was performed using LabChart Pro software (v8.1.3; ADInstruments, Sydney, NSW, Australia). EEG data were processed as hourly averages and presented from 24 h before the first saline or LPS infusion until the end of the experiment. Due to small differences in baseline spectral edge frequency between the group, spectral edge frequency was normalised by subtracting the baseline values (average of 24 h before the first saline/LPS infusion) from the absolute value. EEG data during the baseline, 24 h period after LPS/saline infusion, and recovery (from 24 h after the final LPS infusion until the end of the study, i.e. 72–96 h) periods were analysed separately. Sleep stage cycling was assessed using the raw EEG spectral edge frequency trace during the last 5 h of the experimental period (91–96 h). Sleep stage cycling was defined as a repetitive alternating pattern of high and low-frequency activity, with each phase lasting approximately 20 min, as previously described [35]. Data were tested for normality using the Shapiro–Wilk test. Histological and PCR data were analysed using an unpaired *t*-test. Mann–Whitney *U*-tests were used for testing non-parametric data. For EEG data and Sholl analysis of dendritic morphology, when statistical significance was found between groups, group and time (EEG) or group and radius (Sholl analysis) post hoc comparisons were made using the Fisher’s least significant difference test [36]. Linear and non-linear regression were used to assess the relationship between EEG spectral edge frequency and band power with neuronal microstructure. Post hoc power analysis for summated dendritic length showed 85% power to detect a minimum difference of 970 µm. Statistical significance was accepted when *P* < 0.05. Data are presented as scatter plots with mean ± standard error of the mean (SEM).

## Results

### Baseline period

Before LPS exposure, fetal arterial blood gases, pH, glucose, lactate and EEG power and frequency did not differ between groups and were within the normal range for our laboratory. Blood gas data and EEG power have been previously published in Kelly et al. [24].

### Confirmation of systemic inflammation

All LPS-exposed fetuses had increased plasma cytokine levels relative to baseline after LPS infusions [24], confirming the induction of a systemic inflammatory response.

### Spectral edge frequency (SEF)

**Fig. 2:**
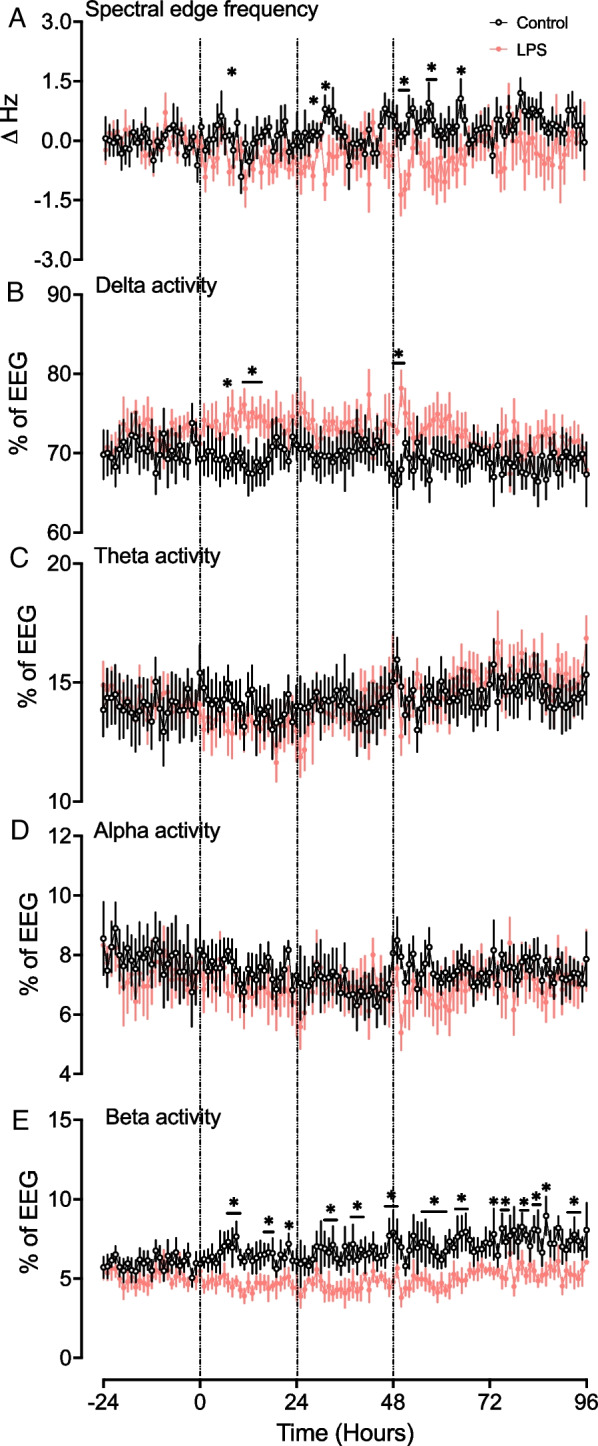
Neurophysiological changes over time. From the top down, the figure shows spectral edge frequency (**A**), %delta activity (**B**), %theta activity (**C**), %alpha activity (**D**), and %beta activity (**E**) in the control (black, *n* = *9*) and LPS (red *n* = *8*) groups. Vertical lines indicate the timings of LPS administration. Data are hourly means ± standard error (SE). *P < 0.05 vs. control

After the first LPS infusion, SEF was lower in the LPS group at 7 h (*P* < 0.05 vs. controls, Fig. 2A). After the second LPS infusion, SEF was reduced in the LPS group from 28–31 h (i.e., 4–7 h after the second infusion; *P* < 0.05 vs. controls). After the third LPS infusion, SEF was reduced in the LPS group from 50–52, 57–58 and at 65 h (i.e., at 2–4 h, 9–10 h and 17 h after the third infusion, *P* < 0.05 vs. controls). Qualitative assessment of EEG spectral edge frequency during the last 5 h of the recording period (91–96 h) showed sleep stage cycling was present in all control and LPS-exposed fetuses.

### Spectral band power analysis

After the first LPS infusion, delta band power increased in the LPS group from 7–15 h (*P* < 0.05 vs. controls, Fig. 2B). After the third LPS infusion, %delta band power was higher in the LPS group from 48–50 h (i.e., 0–2 h after the third infusion, *P* < 0.05 vs controls, Fig. 2B). There were no differences in %theta and %alpha band powers after LPS infusions between groups (Fig. 2C, D).

After the first LPS infusion, %beta band power decreased in the LPS group from 7–9 h and 16–18 h and at 22 h (*P* < 0.05 vs. controls, Fig. 2E). After the second LPS infusion, %beta band power was lower in the LPS group from 31–33 h and 38–40 h and at 47 h (i.e., at 7–9 h, 14–16 h, and 23 h after the second LPS infusion; *P* < 0.05 vs. controls, Fig. 2E). After the third LPS infusion, %beta band power was lower at 48 h and between 55–66 h, 73–86 h, and 92–94 h (i.e., at 0 h, 7–18 h, 25–38 h, and 44–46 h after the third LPS infusion; *P* < 0.05 vs. controls, Fig. 2E).

### Gene expression analysis

**Fig. 3:**
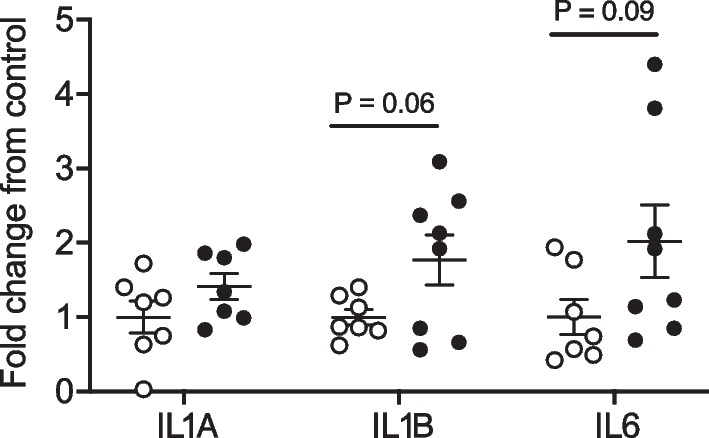
Interleukin (IL)1A, IL1B, and IL6 mRNA levels in the somatosensory cortex in tissue sections adjacent to samples processed for Golgi staining from controls (white circles, *n* = 7, two subjects had undetectable values), and LPS (black circles; IL1B, *n* = 7 [one subject had undetectable values], IL1A, *n* = 8, and IL6, *n* = 8) groups. Data are means ± SE and are expressed as the fold change from the mean control values

There were no significant changes in mRNA expression of IL1A, IL1B (*P* = 0.06) and IL6 (*P* = 0.09) in the somatosensory cortex in the LPS group compared with controls (Fig. 3).

### Neuronal basal dendrite morphology

**Fig. 4:**
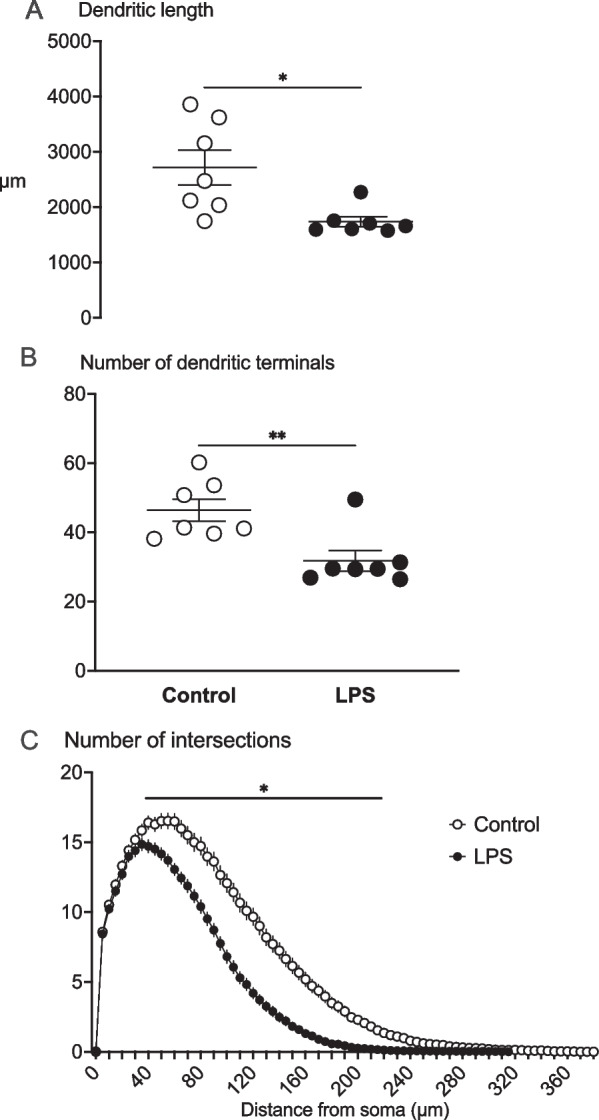
Summated dendritic length (μm) (**A**), number of dendritic terminals (**B**) and Sholl analysis (**C**) showing the number of dendritic intersections (dendritic arborisation) indicated by the number of intersections at 5 μm intervals away from the soma in the control (white circles, *n* = *7*; two subjects had limited Golgi penetration) and LPS (black circles, *n* = *7*; 1 subject had limited Golgi penetration) groups. Data are means ± SE, *P < 0.05 vs control

The summated basal dendritic length of cortical pyramidal neurons was significantly reduced in the LPS group (by ~ 36%) compared to control (control: 2716 ± 314 µm vs. LPS: 1738 ± 92 µm; *P* < 0.05, Figs. 4A, 6A). The number of basal dendritic terminals was significantly reduced in the LPS group (by ~ 31%) compared with controls (control: 46 ± 3 vs. LPS: 32 ± 3, *P* < 0.01, Figs. 4B, 6B). Sholl analysis of pyramidal neuron complexity showed reduced dendritic arborisation in the LPS group at 40–230 μm away from the soma (*P* < 0.05 vs. controls; Fig. 4C).

### Neuronal basal dendritic spine number and morphology

**Fig. 5:**
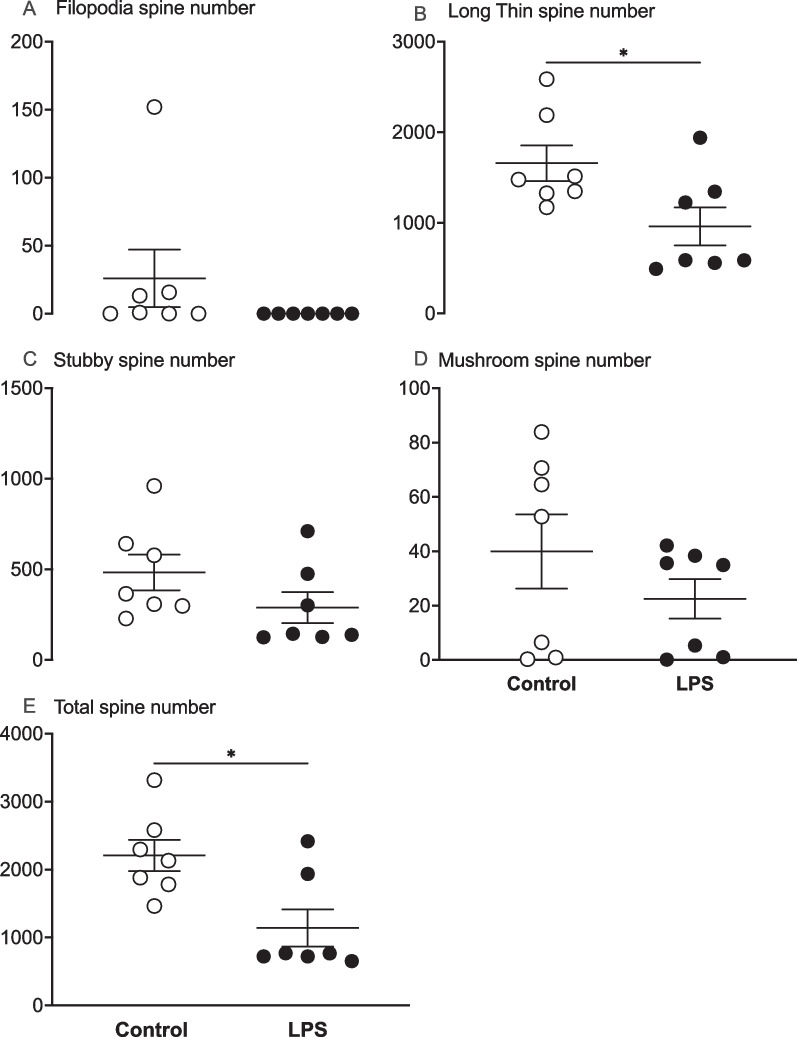
Numbers of filopodia, long thin, stubby, mushroom and total dendritic spines in control (white circles, *n* = *7*; two subjects had limited Golgi penetration) and LPS (black circles, *n* = *7*; 1 subject had limited Golgi penetration) groups. Data are means ± SE, *P < 0.05 vs control

**Fig. 6:**
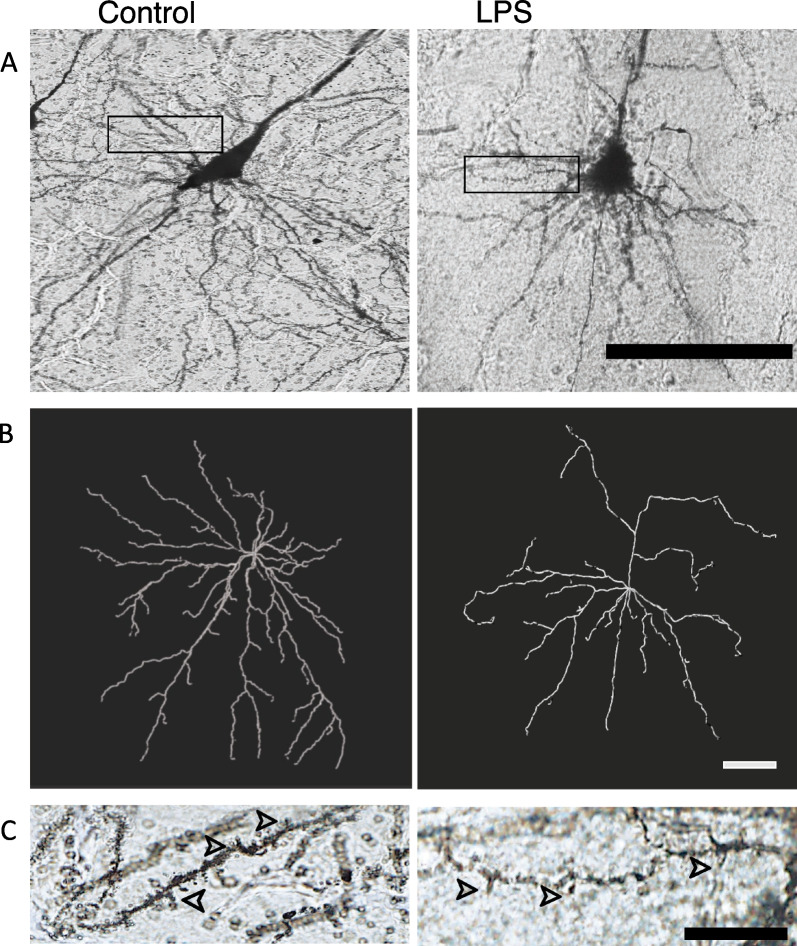
Representative Golgi stained (**A**) and traced (**B**) images of basal dendrites and dendritic spines (**C**) from pyramidal neurons in the somatosensory cortex from control and LPS-exposed subjects. Scale bar panel **A** = 100 μm, panel **B** = 50 μm and panel **C** = 15 μm

The total number of basal dendritic spines was reduced in the LPS group compared with controls (*P* < 0.05, Figs. 5E, 6C). Spine morphology classification showed reduced numbers of long thin spines in the LPS group compared to control (*P* < 0.05; Fig. 5B). There were no differences in numbers of filopodia, stubby or mushroom spines between the groups (Fig. 5A, C, D, respectively).

### Histopathology

**Fig. 7:**
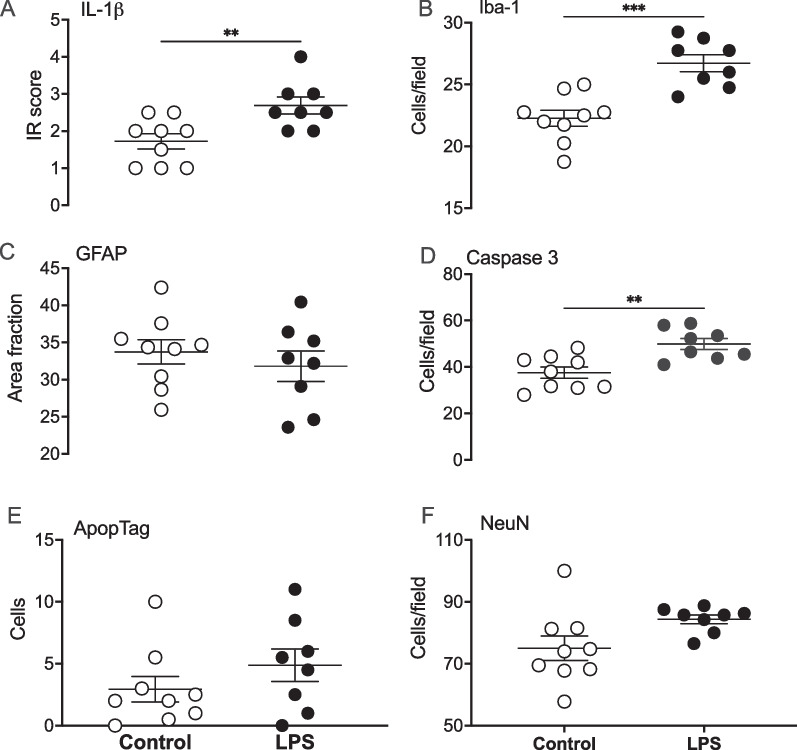
Interleukin 1(IL)-1β immunoreactivity scores (**A**), numbers of ionised calcium binding adaptor molecule (IBA-1) + microglia (**B**), glial fibrillary acidic protein (GFAP +) area fraction staining (**C**), numbers of caspase 3 + cells (**D**), numbers of ApopTag (TUNEL) + cells (**E**) numbers of NeuN + neurons (**F**), in the lateral gyrus (LG) in control (open circles, *n* = *9*) and LPS groups (black circles, *n* = *8*) groups. Data are means ± SE, *P < 0.05 vs control

**Fig. 8:**
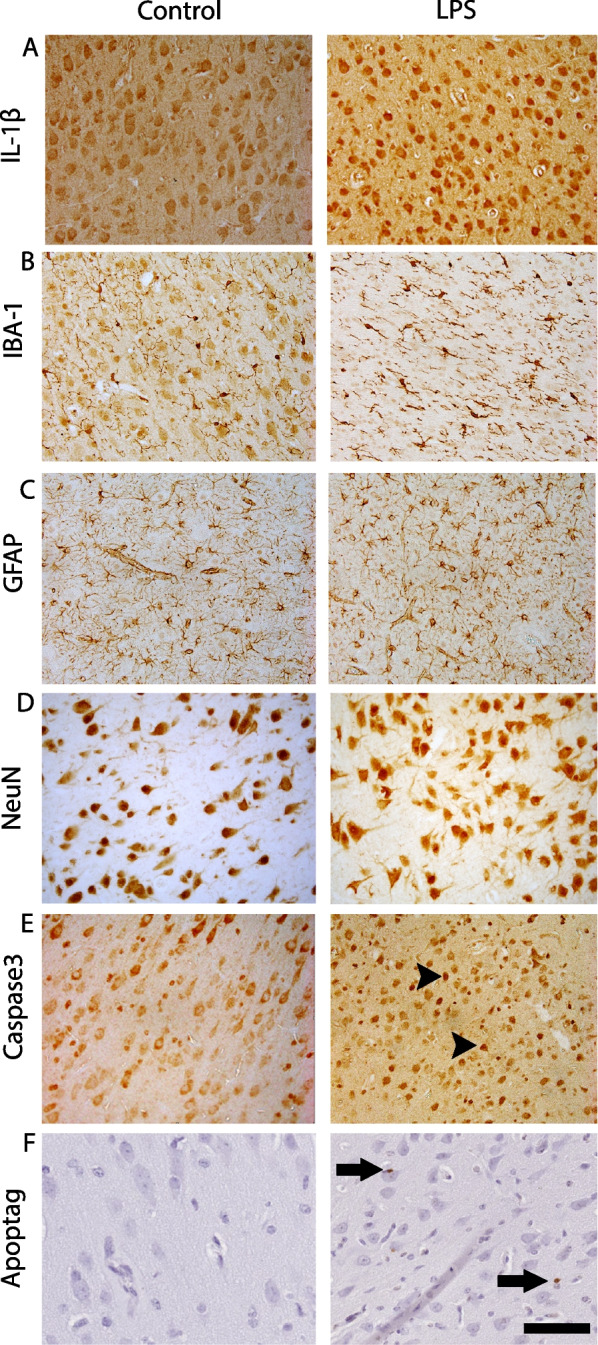
Representative photomicrographs showing immunohistochemical staining of IL-1β (**A**), IBA-1 (**B**), GFAP (**C**), NeuN (**D**), caspase3 (**E**) and ApopTag (TUNEL, **F**) in the lateral gyrus. Arrowheads point to Caspase 3 + cells and arrows point to ApopTag (TUNEL +) cells. Scale bar = 100 μm

Immunoreactivity of IL-1β was increased in the somatosensory cortex of the LPS group (*P* < 0.05 vs. controls; Figs. 7A, 8A). Numbers of Iba-1 + microglia were significantly increased in the LPS group (*P* < 0.01 vs. controls, Figs. 7B, 8B). There was no difference in area fraction of GFAP + astrocyte staining between the groups (Figs. 7C, 8C). Numbers of caspase3 + cells were increased in the LPS group compared with controls (*P* < 0.05; Figs. 7D, 8E). There were no differences in the total numbers of ApopTag + cells between the groups (Figs. 7E, 8F). Finally, there were no differences in either numbers of NeuN + neurons (Figs. 7F, 8D) or cortical area (control: 2.7 × 10^7^ ± 2.4 × 10^6^ µm^2^ vs LPS: 2.6 × 10^7^ ± 3.4 × 10^6^ µm^2^) in the lateral gyrus between the groups.

### Correlative analysis

Mean %beta band power during the final 12 h of the experiment was positively correlated with dendritic length (linear regression: *R*^2^ = 0.30, *p* = 0.0417) and neuronal arborisation (linear regression: *R*^2^ = 0.30, *P* = 0.0423). There were no significant correlations between beta band power and numbers of dendritic terminals and numbers of dendritic spines. There were no significant correlations between spectral edge frequency, or the %delta, %theta and %alpha spectral bands and markers of neuronal microstructure.

## Discussion

This study demonstrates that antenatal inflammation reduced the length, number and complexity (arborisation) of basal dendrites, and reduced the numbers of dendritic spines on pyramidal neurons within the somatosensory cortex in late gestation fetal sheep. The reduction in dendritic length and complexity was associated with an increased number of microglia and IL-1β-positive staining but was not associated with changes in the number of cortical neurons or cortical area. Functionally, the inflammation-induced reduction in dendritic length and complexity were associated with transient increases in delta (slow wave) activity, reduced beta (fast wave) activity and an overall reduction in the spectral edge frequency of the EEG. Given the association between exposure to perinatal inflammation and reductions in cortical growth and connectivity [6, 7, 37], the present study provide critical new insight into the deficits in neuronal structure and function arising from antenatal inflammation.

Clinically, Gram-negative infections, including E. coli, continue to be among the most common pathogens linked to perinatal infection/inflammation and increased risk of perinatal brain injury [38, 39]. We sought to reproduce features of Gram-negative infection/inflammation using repeated increasing doses of LPS infusions to promote a chronic progressive fetal inflammatory response that is commonly associated with adverse neurodevelopmental outcomes [24, 40]. By contrast, most of the previous preclinical studies have focused on the pathophysiological consequences of single or repeated bolus doses of LPS or other infectious/inflammatory stimuli [41].

This study showed that inflammation induced by increasing doses of LPS infusions was associated with increased numbers of microglia and greater immunoreactivity of IL-1β in the somatosensory cortex but no significant increases in cortical IL1B (*p* = 0.06), IL6 (*p* = 0.09) or IL1A mRNA expression. Consistent with this observation, human post-mortem studies have reported increased numbers of microglia and IL-1β immunoreactivity in areas of white and grey matter inflammation and injury [42–44]. Indeed, cerebral recognition of pathogen-associated molecular patterns such as LPS by innate immune receptors, including toll like receptor 4, on microglia and other immune cells leads to glial cell activation and nuclear factor kappa B induced transcription of bioactive IL-1β [45]. Furthermore, circulating cytokines, including IL-1β, can penetrate the blood brain barrier [46–48] to recruit and activate microglia within the central nervous system [45]. Thus, the concomitant increase in numbers of microglia and IL-1β immunoreactivity observed in this study strongly supports sustained inflammation in the somatosensory cortex 4 days after beginning intravenous LPS infusions.

In this experimental model of antenatal inflammation, we have previously reported progressive increases in concentrations of systemic pro- and anti-inflammatory cytokines (IL-1β, tumour necrosis factor [TNF], IL-6 and IL-10), in addition to diffuse white matter gliosis and reduced numbers of precursor oligodendrocytes [24]. Clinically, in large prospective studies of preterm infants, increased concentrations of these inflammatory proteins in cord blood and postnatal blood samples have been associated with perinatal brain injury and impaired neurodevelopment in childhood [49]. The present study shows that exposure to inflammation did not affect overall numbers of neurons (NeuN +) or neuronal density in the areas of the somatosensory cortex evaluated in this study, suggesting a lack of overt cortical injury. This is further confirmed by the similar numbers of TUNEL + cells between groups, suggesting no effect of LPS-exposure on acute cell death in the somatosensory cortex. This observation is consistent with neonatal experimental and clinical studies showing limited or no neuronal cell death in cases of perinatal encephalopathy, including after systemic inflammation [10, 49, 50]. By contrast, we observed increased numbers of caspase 3 + cells in LPS-exposed fetuses compared to controls. This finding of increased numbers of caspase-3-positive cells without increased cell death has been reported in the adult and perinatal brain [51–54] and is likely to be linked to other roles played by caspases, which include immune/microglial activation and cell differentiation [54–56].

We observed a reduction in neuronal dendritic complexity in LPS-exposed fetuses as shown by reduced dendritic length, numbers of dendritic terminals, dendritic arborisation, and numbers of dendritic spines. In humans, the marked cortical expansion that occurs during the last trimester primarily reflects the prolific increase in neuronal dendritic growth and complexity during this stage of development [57, 58]. Our observations suggest that at this period in late gestation, neuronal development within the somatosensory cortex in the developing fetus is highly vulnerable to inflammation-induced impairments of dendritic arborisation and spine formation. These data are consistent with previous studies that reported reduced neuronal arborisation in the frontal cortex of fetal sheep after acute cerebral ischaemia at mid-gestation, and reduced dendritic number and spine density in the retrosplenial cortex of newborn rabbits (P1) after a single bolus of intra-amniotic LPS (20 µg/kg) [59]. Similarly, in separate rodent studies examining the long-term effects of prenatal and early postnatal LPS-induced inflammation, reduced dendritic arborisation was seen in the motor cortex and medial prefrontal cortex on postnatal days 21 and 60 [10, 60]. Furthermore, these data support a link between diffuse white matter injury, which we have previously reported in the same experimental paradigm [24], and impaired neuronal development. For example, human case series have shown reduced dendritic length in cases of both diffuse and necrotic white matter injury [61, 62]. Similarly, moderate LPS-induced inflammation in neonatal rodents (from P1-P3) was associated with reduced dendritic arborisation in the motor cortex, diffuse white matter injury, and impaired myelination and motor function on postnatal day 21 [10].

The somatosensory cortex has been shown to synapse with cervical excitatory neurons and modulate locomotion independently of the motor cortex [63]. Consistent with this observation, we have previously reported reduced fetal movements in the same fetal sheep paradigm, as shown by reduced nuchal electromyographic activity from 3 days after starting LPS infusions until the time of post-mortem [24]. Although we did not evaluate neuronal complexity within the motor cortex in this study, our data raise the possibility that impaired neuronal development in the somatosensory cortex may contribute to the inflammation-induced reduction in fetal body movements observed in this preclinical model of antenatal infection/inflammation. Taken together, these data support a close link between impaired neuronal development in the somatosensory cortex and inhibition of motor function.

Although the precise mechanism/s underpinning the inflammation-induced impairment in neuronal development are yet to be identified, it is likely to include a direct effect of inflammation on the central nervous system. For example, in vitro studies have shown that cortical neurons exposed to inflammatory cytokines, including IL-1β, IL-6, TNF, and interferon gamma, show reduced dendritic branching and synapse formation [64, 65]. This is supported by our findings of increased numbers of cortical microglia, which are known to secrete proinflammatory cytokines, along with increased IL-1β immunoreactivity observed in the LPS-exposed fetuses. Microglial processes have also been shown to interact with synapses to eliminate spines, suggesting a direct effect of microglial activation on spine density [66, 67]. Furthermore, reduced circulating concentrations of neural growth factors, including nerve growth factor and brain derived neurotrophic factor, have been reported in human and animal studies of perinatal infection/inflammation [59, 68, 69].

Consistent with the inflammation-induced reduction in neuronal complexity in the present study, neuronal activity in LPS-exposed fetuses was impaired, as shown by an overall reduction in the spectral edge frequency of EEG activity along with an increase in the proportion of EEG activity in the delta band and a reduced proportion of activity in the beta band. Collectively, these data indicate loss of high-frequency activity after LPS-exposure with a shift to lower frequency activity. The inflammation-induced change in EEG spectra could reflect alterations to fetal behaviour/sleep stages. The fetus is never awake, but rather cycles between low voltage (high frequency) and high voltage (low frequency) sleep [70]. Qualitative analysis of EEG frequency signals over the last 5 h of the experimental period showed sleep stage cycling was present in all control and LPS-exposed fetuses. Consistent with these data, pre- and post-natal immune challenges in mice have shown inflammation-induced increases in slow wave sleep in association with a shift in spectral band power (increased low-frequency and reduced high-frequency activity) that was consistent with our analysis [71]. Although sleep stage cycling was observed in all fetuses in the present study at the end of the recording period, immediately prior to post-mortem, it is possible that the inflammation-induced changes in EEG spectra are associated with subtle changes in the proportions of high and low-frequency sleep.

We have previously reported that there were no differences in myelin density or numbers of mature myelinating oligodendrocytes at this timepoint in this cohort of LPS-exposed fetuses [24]. This suggests that the changes in EEG frequency in the present study are not related to altered myelination. Alternatively, the inflammation-induced reduction in high-frequency activity may reflect inhibition of synaptic activity. This could be due to the reduced neuronal arborisation and numbers of dendritic spines on cortical neurons directly underlying the EEG electrodes (i.e., a direct functional consequence of inflammation-induced changes in neuronal pathology). Alternatively, elevated central levels of IL-1β have been shown to induce NMDA-mediated suppression of synaptic function [72]. Consistent with this, TNF inhibition using the soluble TNF receptor antagonist, etanercept, reduced the magnitude of EEG suppression in fetal sheep exposed to LPS [73], possibly due to reduced NMDA receptor activation [74]. Furthermore, in vivo and in vitro studies have shown that both LPS- and IL-1β-induced central inflammation actively mediate EEG suppression through the release of inhibitory neuromodulators, such as allopregnanolone and adenosine [75, 76]. Taken together, the present data suggest that inflammation-induced suppression of EEG activity is mediated by a combination of activation of anti-excitotoxic mediators as well as reduced complexity of the neuronal microstructure.

Clinical studies have shown that reduced EEG frequency strongly predicts subsequent brain injury and neurodevelopmental impairment in preterm and term infants. For example, in a cohort study, reduced EEG frequency was associated with the severity of neonatal white matter injury [77]. Similarly, depression of the EEG background pattern was associated with both motor and cognitive impairment in preterm and term infants with evidence of central inflammation [78, 79]. Furthermore, increased latency of somatosensory evoked potentials was reported in children with bilateral spastic cerebral palsy. Notably, the latency of somatosensory evoked potentials correlated with a history of exposure to perinatal infection/inflammation [80]. Functional MRI studies have shown reduced cortical functional connectivity in fetuses exposed to inflammation before birth [37]. Similarly, reduced cortical functional connectivity was observed in preterm infants without evidence of overt cortical injury [81]. Subsequent investigation of infants with moderate to severe white matter injury, but without overt cortical injury, showed a reduction in cortical functional connectivity that correlated with the severity of white matter injury [82]. Our data suggest that in the absence of overt neuronal injury or white matter loss, reduced neuronal complexity and synaptic density may contribute to reduced functional connectivity within and between major grey matter structures after exposure to perinatal inflammation.

This study was not designed to test the effect of sex and is not large enough to determine whether there were sex-specific effects of antenatal inflammation on neuronal microstructure or EEG activity. Previous studies in perinatal rodents reported sex-dependant effects in central and peripheral immune activation, as well as neuronal development [83, 84]. Further studies evaluating the impact of sex on inflammation-induced changes to neuronal structure and function in large animals are needed.

In conclusion, this study demonstrates that an inflammation-induced reduction in high-frequency spectral band power was associated with reduced cortical neuronal arborisation and dendritic spine density. Collectively, these data support the concept that inflammation-induced impairments in neuronal maturation and function, rather than overt neuronal loss, make a key contribution to disturbed cortical development and connectivity, and subsequent impaired neurodevelopmental outcomes, in infants exposed to perinatal inflammation. We propose that early EEG monitoring combined with neuroimaging modalities that enable more sensitive assessment of brain microstructure [10, 85] and therapeutics designed to mitigate systemic and central inflammation, could provide an effective approach for early detection and therapeutic intervention.

## Data Availability

The datasets used during the current study are available from the corresponding author upon reasonable request.
